# Limbic Encephalitis: Potential Impact of Adaptive Autoimmune Inflammation on Neuronal Circuits of the Amygdala

**DOI:** 10.3389/fneur.2015.00171

**Published:** 2015-08-03

**Authors:** Nico Melzer, Thomas Budde, Oliver Stork, Sven G. Meuth

**Affiliations:** ^1^Department of Neurology, University of Münster, Münster, Germany; ^2^Institute of Physiology I, University of Münster, Münster, Germany; ^3^Department of Genetics and Molecular Neurobiology, Institute of Biology, Otto-von-Guericke University Magdeburg, Magdeburg, Germany; ^4^Department of Neuropathophysiology, Institute of Physiology I, University of Münster, Münster, Germany

**Keywords:** amygdala, circuit, autoimmunity, limbic encephalitis, T cells, B cells, antibodies, neurons

## Abstract

Limbic encephalitis is characterized by adaptive autoimmune inflammation of the gray matter structures of the limbic system. It has recently been identified as a major cause of temporal lobe epilepsy accompanied by progressive declarative – mainly episodic – ­memory disturbance as well as a variety of rather poorly defined emotional and behavioral changes. While autoimmune inflammation of the hippocampus is likely to be responsible for declarative memory disturbance, consequences of autoimmune inflammation of the amygdala are largely unknown. The amygdala is central for the generation of adequate homoeostatic behavioral responses to emotionally significant external stimuli following processing in a variety of parallel neuronal circuits. Here, we hypothesize that adaptive cellular and humoral autoimmunity may target and modulate distinct inhibitory or excitatory neuronal networks within the amygdala, and thereby strongly impact processing of emotional stimuli and corresponding behavioral responses. This may explain some of the rather poorly understood neuropsychiatric symptoms in limbic encephalitis.

## Introduction – Autoimmune Inflammation of the Amygdala in Limbic Encephalitis

Limbic encephalitis is characterized by adaptive autoimmune inflammation of the gray matter structures of the limbic system (1). It has recently been identified as a major cause of temporal lobe epilepsy accompanied by progressive declarative – mainly episodic – memory disturbance as well as a variety of rather poorly defined emotional and behavioral changes (2–4).

Magnetic resonance imaging exhibits dynamic changes of volume and signal intensity that use to be most prominent in the amygdala and the hippocampus suggesting considerable inflammation and subsequent degeneration (together with structural reorganization) in these brain areas (1, 5–7). While autoimmune inflammation of the hippocampus is likely to be responsible for declarative memory disturbance, consequences of autoimmune inflammation of the amygdala are poorly understood (8).

A role of the amygdala in appropriate human emotional behavior was first reported by Adolphs et al. (9). This finding was supported by studies on individuals with impaired ability to recognize emotions from facial or prosodic expressions after amygdala damage of variable etiology (10–12). However, the recognition of facial (13) and prosodic (14, 15) emotions is apparently normal in some patients with amygdala damage. Two factors related to this discrepancy have been suggested: (i) age at damage onset [early-onset (congenital) vs. late-onset of amygdala damage] and (ii) extent of the damage (selective amygdala damage vs. broad damage to the mesial temporal lobe) (16).

Emotion recognition (happiness, sadness, anger, fear, surprise, disgust) from facial and non-facial stimuli was investigated in two patients with non-herpetic limbic encephalitis 14 weeks after the onset of the disease. One patient who had a lesion relatively restricted to the amygdala and hippocampus experienced difficulty in recognizing fear from facial expressions. In contrast, the other patient who had a lesion that extended beyond the amygdala experienced difficulty in recognizing fear from non-facial (prosodic and written verbal) stimuli (17). Moreover, recognition of emotional stimuli, such as fear, and disgust both from faces and voices has been shown to be impaired in a patient with Ma antibody-associated limbic encephalitis (18).

Furthermore, autonomic responses to such emotional stimuli have been reported to be severely impaired in patients with limbic encephalitis. Sweating on the palms of the hands and soles of the feet, so-called emotional sweating, is considered to be mediated by the limbic system, including the amygdala and anterior cingulate cortex. Hence, sweat and skin vasoconstriction responses to arousal stimuli were recorded on the palms of seven patients with viral (herpes simplex virus and Epstein–Barr virus encephalitis; *n* = 3) and autoimmune (voltage-gated K^+^ channel antibody positive, glutamate receptor antibody positive, and antibody-negative limbic encephalitis; *n* = 4) bilateral limbic encephalitis, which included both the amygdala and hippocampus 3 weeks to 4 months after disease onset. Sweat responses and skin vasoconstriction responses were absent or markedly reduced in patients with limbic encephalitis compared to normal controls following a variety of emotional stimuli (19). The same results were obtained in a patient with autoimmune limbic encephalitis restricted bilaterally to the amygdala (20) indicating that affection of the amygdala rather than the hippocampus seemingly accounts for impaired emotional sweating in these patients.

Interestingly, another case of extensive bilateral limbic damage (including amygdala damage) after an episode of herpes simplex virus encephalitis 30 years ago did not present any emotional impairment besides difficulty to identify anger expression (21), suggesting that some adaptive mechanisms may compensate for the neuropsychological symptoms during the disease course.

The amygdala is an almond-shaped nucleus located deep in the temporal lobe. It is considered as a core region of the limbic system involved in the control of positive and negative affects, as well as the modulation of social and cognitive functions (22). Different amygdala subnuclei exist, of which we will focus here on lateral (LA) and basal amygdala (BA), which are often jointly (together with the accessory-basal nuclei) considered the basolateral complex of the amygdala (BLA). We argue that interference with BLA function through known B cell-derived autoantibodies and T cells may explain, at least in part, some neuropsychiatric features in limbic encephalitis.

The BLA is a main input site for sensory information reaching the amygdala from thalamic and cortical regions and for their convergence with affective information. Sensory input is organized in a topographic manner in that the LA is more concerned with unimodal information, whereas the BA tends to receive more complex multimodal inputs. Through its projections to the central amygdala (CeA), the bed nucleus of the stria terminalis (BNST), and the ventral hippocampus (23, 24), the BLA has been implicated in the control of fear and anxiety [for review see Ref. (25)]. Moreover, the BLA modulates reward and addiction via direct projections to the nucleus accumbens (26) and controls the function of the medial prefrontal cortex (27).

The amygdala receives prominent neuromodulatory input and triggers arousal and stress responding through projections to the septum and locus coeruleus. It is further a key site for the activity of corticosterone in the brain, which increases excitability of BLA projection neurons and reduces the inhibition through γ-aminobutyric acid (GABA) receptors (28). By mediating the effects of these modulatory factors, the BLA is also thought to act as an important modulator of neural plasticity and memory formation in the hippocampus and prefrontal cortex (29, 30).

**Figure 1:**
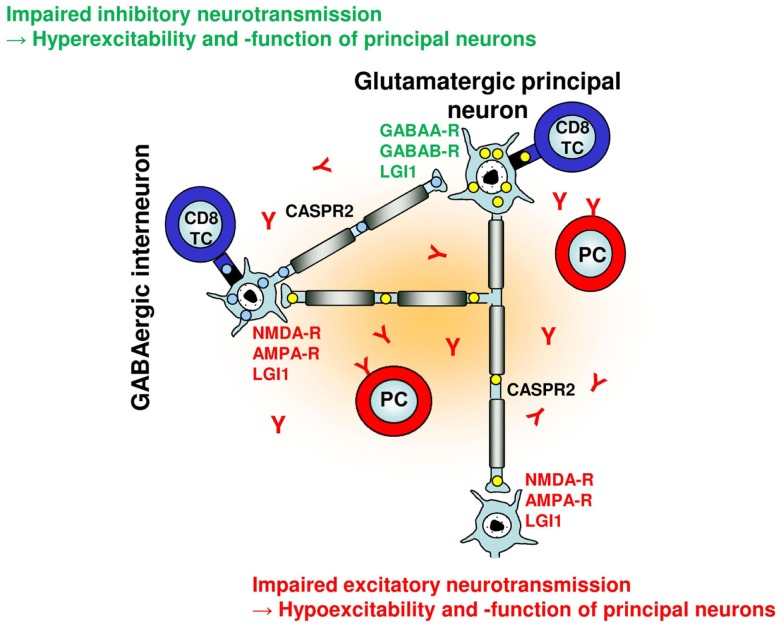
**Putative effects of adaptive humoral and cellular autoimmunity on a simplified neuronal network**. Glutamatergic principal neurons and GABAergic interneurons can be selectively targeted by neuronal antigen-specific CD8^+^ T cells based on their differential *intracellular* antigen expression (and presentation) [e.g., GAD65 in interneurons (blue), Hu in principal neurons (yellow)] with distinct consequences for network function and excitability. With regard to neuronal cell membrane antigens, excitatory glutamatergic synaptic transmission and plasticity can be disturbed by antibodies against NMDA and AMPA receptors, GABAergic synaptic transmission and plasticity can be disturbed by antibodies against GABA_A_ and GABA_B_ receptors. Antibodies against LGI1 and CASPR2 may interfere with both glutamatergic and GABAergic synaptic transmission and intrinsic neuronal excitability within the network, respectively.

Glutamatergic principal cells in the BLA are under a tight control by GABAergic interneurons (Figure 1). Approximately 10–20% of neurons in the amygdala are GABAergic and control information flow as well as rhythmic network activity (31). Various types of inhibitory interneurons (32) exist in the BLA that control specific aspects of information flow and behavioral function (33). Disinhibitory local circuits have also been reported (34) and implicated in fear memory formation (35). This internal circuitry of the BLA is particularly well set to process information via rhythmic network activities. BLA neurons show intrinsic low-frequency oscillations (36) and can develop gamma oscillations upon GABAergic blockage (37). GABAergic interneurons are furthermore selectively recruited to theta rhythms originating in the hippocampus (38). Thus, network oscillations in the BLA closely reflect periods of safety and threat exposure (39) and synchronize with the hippocampus and prefrontal cortex during states of fear retrieval and extinction (40, 41).

Amygdala lesions lead to emotional numbness and fearlessness, whereas hypertrophy of amygdala has been observed in patients with post-traumatic stress disorder and depression (42). Indeed, it has been suggested that the amygdala is a key structure for the long-term behavioral adaptation to stress (43). The amygdala has furthermore been identified as a major epileptic focus in temporal lobe epilepsy, and in rodents it is widely used as a site of stimulation in the kindling model of epilepsy. Importantly, within the amygdala the BLA plays a central role in seizure generation (44).

Considering these findings, we hypothesize that changes in BLA excitability and information processing induced by autoimmune inflammation contribute to seizures, different levels of anxiety, mood disorder, and potentially also memory deficits in limbic encephalitis as – depending on the predominant immune effector mechanism and the neuronal target antigen – autoimmune inflammation of the amygdala may result in decreased or increased excitability and function of principal neurons of the BLA network (Figure 1; Table 1).

**Table 1:** **Putative effects of adaptive humoral and cellular autoimmunity on inhibitory and excitatory transmission and network activity of the amygdala together with potential clinical consequences**.

	Immune mechanisms	Target antigens	Neuronal effects	Potential clinical effects
Inhibitory neurotransmission	Humoral	GABA_A_ receptor abs	Hyperexcitability and function of principal cells	State of increased anxiety, generalized fear and hyperarousal, epileptic seizures
		GABA_B_ receptor abs		
		LGI1 abs		
		CASPR2 abs		
	Cellular	GAD65-reactive T cells and others	Hyperexcitability and function of principal cells	State of increased anxiety, generalized fear and hyperarousal, epileptic seizures
Excitatory Neurotransmission	Humoral	NMDA receptor abs	Hypoexcitability and function of principal cells	Disturbed processing of emotional stimuli, lower levels of anxiety, generalized fear
		AMPA receptor abs		
		LGI1 abs		
		CASPR2 abs		
	Cellular	HuD-reactive T cells and others	Hypoexcitability and function of principal cells	Disturbed processing of emotional stimuli, lower levels of anxiety, generalized fear

*For details, please refer to the text*.

## Putative Pathogenesis of Autoimmune Gray Matter Inflammation and Consequences for Neuronal Function and Integrity

Adaptive neuron-directed autoimmunity underlying limbic encephalitis is illustrated by the presence of specific anti-neuronal antibodies binding to either intracellular or plasma membrane neuronal antigens in sera and cerebrospinal fluid in many cases (45–47). Depending on the cellular localization of their antigens, these antibodies provide some hints on the predominant autoimmune effector mechanisms toward single neurons and neuronal networks.

In a subgroup of patients with limbic encephalitis, autoantibodies are detected that bind to *intracellular neuronal* *antigens*. These are molecules with a role in a variety of gene expression and signal transduction processes, which are expressed in distinct neuronal cell populations [reviewed in Ref. (46)]. In these patients, CD8^+^ T cells usually recognize continuous linear peptide epitopes consisting of 8–10 amino acids that are derived from intracellular neuronal proteins by extensive antigen processing and presented in the context of MHC I molecules on the cell surface membrane (48–51). T cell receptor (TCR)-signaling upon recognition of the appropriate antigen in the context of MHC I molecules during engagement of activated CD8^+^ T cells with neurons leads to the formation of the immunological synapse (52). Subsequent CD8^+^ T cell-mediated impairment of neuronal excitability and integrity is predominantly mediated via two largely independent pathways (53, 54). (i) Granule cytotoxicity occurs by liberation of perforin together with a variety of granzymes. Depending on the amounts released, perforin alone can lead to the formation of unselective transmembrane pores with a subsequent impairment of electrical excitability and signaling (50, 55) or neuronal necrosis with immediate swelling and rupture of the neuronal cell membrane (56). Alternatively, perforin mediates the trafficking of granzymes into the target cell promoting apoptosis within few hours (56, 57). (ii) Impairment of neuronal excitability and structural integrity may also occur through the ligation of cell death receptors (50, 58). Moreover, CD8^+^ T cells are able to liberate potentially neurotoxic cytokines, such as interferon-(IFN)γ and tumor necrosis factor-(TNF)α (59, 60) as well as distinct neurotransmitters like glutamate (61, 62), adding to the repertoire of molecular effector mechanisms directly impacting on neuronal excitability and integrity (63).

Given these molecular effector mechanisms, CD8^+^ T cells cannot directly impact the function or expression of their cognate neuronal antigens but recognize their expression by the respective neuron. This enables them to contribute to neuronal dysfunction and cell death (50, 55, 64, 65). These effects may be restricted to distinct inhibitory (1) or excitatory (66, 67) neuronal populations and networks due to their differential antigen expression pattern and capability of MHC I-mediated antigen presentation (65, 68).

In a subset of patients with limbic encephalitis, antibodies against a family of intracellular neuron-specific RNA binding proteins, the neuronal “embryonic lethal and abnormal vision-like” RNA binding proteins, HuB, HuC, and HuD, can be detected (69, 70). These Hu proteins are expressed in specific neuronal populations, including large glutamatergic pyramidal-like neurons in layer V of the neocortex, the cornu amonis (CA) 1–4, and dentate gyrus (DG) regions of the hippocampus, as well as principal cells of the amygdala (71). Neuron-specific Hu proteins have recently been shown to maintain neuronal glutamate levels by stabilizing glutaminase mRNA and protein levels (66, 67). Glutamate is the major excitatory neurotransmitter in the mammalian brain and also impacts inhibitory neuronal signaling in two ways: (i) it is the biochemical precursor for the major inhibitory neurotransmitter GABA (72) and (ii) activates inhibitory neuronal feedback loops (73). Consistent with a net inhibitory effect of Hu-expressing neurons on neuronal network excitability, genetic deficiency of neuron-specific Hu proteins leads to spontaneous neuronal hypersynchrony and epileptic seizure activity (66, 67). Moreover, Hu-expressing neurons have been implicated in hippocampus and amygdala-based synaptic plasticity, learning, and memory (74, 75).

In another subset of patients with limbic encephalitis, antibodies against neuronal glutamate decarboxylase (GAD) have been detected (1). GAD is an intracellular enzyme expressed in a subset of interneurons and catalyzes the conversion of glutamate to GABA therein. The brain contains two isoforms, GAD65 and GAD67, which display characteristic differences in localization and activity patterns (76, 77). GAD67 is typically distributed throughout the neuron and almost all of it exists in its active cofactor-bound form, whereas GAD65 is predominantly found in synaptic terminals and much of it is in the form of an inactive apoenzyme (72, 78). In accordance with a net inhibitory effect of GAD65-expressing interneurons on neuronal network excitability, genetic deficiency of GAD65 leads to spontaneous neuronal hypersynchrony and epileptic seizures (79). Moreover, GAD65-expressing neurons have been implicated in hippocampus and amygdala-based synaptic plasticity, learning, and memory (41, 80–82).

In light of these physiological effects of Hu-expressing pyramidal-like neurons and GAD65-expressing interneurons on synaptic transmission, plasticity, learning, and memory, as well as neuronal network excitability and seizure development, it seems conceivable that similar effects observed upon genetic deficiency of these ­molecules occur if these cell populations become targets of neuronal antigen-specific CD8^+^ T cells as in limbic ­encephalitis (51).

In another subgroup of patients with limbic encephalitis, autoantibodies are detected that bind to *synaptic and extrasynaptic neuronal cell membrane antigens*. These include a variety of ionotropic and metabotropic neurotransmitter receptors and associated molecules [reviewed in Ref. (46)]. In these patients, antibody-mediated disturbance of synaptic transmission and plasticity as well as neuronal (network) excitability occurs together with some neurodegenerative effects (51).

Antibodies recognize and bind to discontinuous conformational epitopes composed of segments of the respective neuronal plasma membrane protein antigen that come in close spatial proximity in their three-dimensional structure and exposed on the neuronal plasma membrane. These are synaptic and extra-synaptic ligand- and voltage-gated ion channels involved in excitatory [mainly *N*-methyl-d-aspartate (NMDA) (83, 84) and α-amino-3-hydroxy-5-methyl-4-isoxazolepropionic acid (AMPA) (85, 86) receptors] and inhibitory [mainly GABA_A_ (87) and GABA_B_ (88) receptors] synaptic transmission and plasticity. Moreover, these antibodies also target neuronal membrane proteins implicated in clustering of voltage- and ligand-gated ion channels inside the synapse [leucine-rich glioma-inactivated 1; LGI1 (89, 90)] or outside the synapse at the juxtaparanodal region of the node of Ranvier [contactin-2 and contactin-associated protein-like 2; CASPR2 (89, 91)], thereby indirectly impacting neuronal excitability.

Depending on the IgG subtype, antibodies may (i) specifically activate or block the function of their target molecules, (ii) crosslink and internalize the receptors, (iii) activate the complement cascade with subsequent formation of the terminal membrane attack complex and target cell lysis, and (iv) activate Fc-receptors with subsequent antibody-dependent cell-mediated cytotoxicity (ADCC) mainly by NK cells (92).

Regarding excitatory synaptic transmission, NMDA receptor antibodies have been described to directly impact the gating behavior of the receptor (93). It has been shown that the N368/G369 region of the GluN1 subunit of NMDA receptors may represent the immunodominant binding region for IgG on the receptor molecule. In single channels recordings, antibody binding to the receptor caused more frequent channel openings and prolonged open time of the receptor as immediate effects (93). Moreover, NMDA receptor antibodies have been shown to cause a selective and reversible decrease in post-synaptic NMDA receptor surface density and synaptic localization in inhibitory as well as excitatory cultured rat hippocampal neurons by selective antibody-mediated cross-linking and internalization (94, 95). Consistently, NMDA receptor antibodies selectively decreased NMDA receptor-mediated miniature excitatory post-synaptic currents (mEPSCs) without affecting AMPA receptor-mediated mEPSCs in cultured rat hippocampal neurons. However, despite these strong effects, NMDA receptor antibodies did not impact the number of synapses, dendritic spines, dendritic complexity, or cell survival in cultured rat hippocampal neurons (94). Consistent with these mechanisms, NMDA receptor antibodies have been shown to suppress induction of long-term potentiation (LTP) at Schaffer collateral-CA1 synapses in mouse hippocampal slices (96). Once internalized, antibody-bound NMDA receptors traffic through both recycling endosomes and lysosomes, but do not induce compensatory changes in glutamate receptor gene expression. This process eventually results in a decrease in inhibitory synapse density onto excitatory cultured rat hippocampal neurons through distinct homeostatic synaptic plasticity ­mechanisms (95, 97).

Additionally, autoantibodies in limbic encephalitis target GluA1 and GluA2 subunits of the AMPA receptor (85, 98). Application of antibodies to cultures of neurons reversibly decreased the number of GluA1- and GluA2-containing AMPA-receptor clusters at synapses and beyond through increased internalization and degradation of surface AMPA receptors (85, 98). In contrast, antibodies do not alter the density of excitatory synapses or cell viability (98). Consistently, whole-cell patch clamp recordings of cultured hippocampal neurons incubated with antibodies revealed decreased AMPA receptor-mediated currents, but not NMDA receptor-mediated currents. Interestingly, probably by distinct homeostatic plasticity mechanisms like those observed by NMDA receptor antibodies (97), several functional properties of AMPA receptor antibody-targeted neurons are altered. Affected neurons exhibit decreased inhibitory post-synaptic currents (IPSCs) and reduced inhibitory synapse density onto excitatory cultured rat hippocampal neurons, whereas the intrinsic excitability of neurons and short-interval firing increase (98).

Hence, both NMDA and AMPA receptor antibodies eliminate ionotropic glutamate receptors from the post-synaptic (and extra-synaptic) neuronal plasma membrane through cross-linking and internalization, resulting in disturbed excitatory synaptic transmission and a concomitant homeostatic decrease in inhibitory synaptic transmission and increased intrinsic excitability.

Antibodies to LGI1 have been described in limbic encephalitis (89, 90). Extracellularly secreted LGI1 has been reported to link two receptors, ADAM (a disintegrin and metalloproteinase domain-containing protein) 22 and ADAM23, and establish a transsynaptic protein complex that includes presynaptic voltage-gated K^+^ channels and post-synaptic AMPA receptors. A lack of LGI1 disrupts this synaptic protein connection and selectively reduces AMPA receptor-mediated synaptic transmission in the hippocampus (99, 100). LGI1 antibodies associated with limbic encephalitis specifically inhibit the ligand–receptor interaction between LGI1 and ADAM22/23 by targeting the EPTP repeat domain of LGI1 and reversibly reduce synaptic AMPA receptor clusters in rat hippocampal neurons (101). Interestingly, conditional knockout of LGI1 restricted to glutamatergic pyramidal cells is sufficient to generate seizures, whereas seizure thresholds were shown to be unchanged after depletion of LGI1 in GABAergic interneurons. Hence, LGI1 secreted from excitatory pyramidal neurons, but not inhibitory interneurons, makes a major contribution to LGI1-related epileptogenesis (102). Similar effects may be expected from antibody-mediated disruption of LGI1 function.

Antibodies against contactin-2 and CASPR2 (89, 91) have been implicated to impair clustering of voltage-gated K^+^ channels at the juxtaparanodal region of the node of Ranvier of myelinated axons, thereby probably interfering with axonal excitability and action potential conduction.

Regarding inhibitory synaptic transmission, antibodies directed against the extracellular epitope of the β3 subunit of the GABA_A_ receptor have been reported (87). Rat hippocampal neuronal cultures exposed to GABA_A_ receptor antibodies specifically decreased both synaptic and surface GABA_A_ receptors, and showed selectively reduced miniature inhibitory post-synaptic currents (mIPSCs) without affecting mEPSCs (103). Concomitant changes in excitatory synaptic transmission have not been reported thus far.

The presence of GABA_B_ receptor autoantibodies constitutes another form of limbic encephalitis (88). GABA_B_ receptors are G-protein-coupled receptors composed of two subunits, GABA_B1_ and GABA_B2_ (104). The main antigen recognized by the antibodies, the GABA_B1_ subunit, is necessary for GABA binding and receptor function, whereas the GABA_B2_ subunit is required for localization of the receptor to appropriate areas of the cell membrane and G-protein coupling (104). GABA_B_ receptors mediate presynaptic inhibition by activation of G-protein-coupled-inward rectifying K^+^ channels and inhibition of Ca^2+^ channels. Post-synaptic GABA_B_ receptors mediate inhibition by similar mechanisms and by inducing a slow inhibitory post-synaptic potential (104). GABA_B_ receptors limit the duration of increased activity states in neuronal networks, preventing excessive neuronal synchronization, and development of epileptic seizures. Hence, although experimental evidence of the molecular consequences of antibody targeting of GABA_B_ receptors is still lacking, it seems conceivable to assume that they will also reduce pre- and post-synaptic GABAergic inhibition and thus promote increased activity states with excessive synchronization in neuronal networks and promotion of epileptic seizures.

Taken together, experimental evidence thus far suggest reduced inhibitory synaptic transmission (and plasticity) – either indirectly via homeostatic plasticity mechanisms following antibody binding to NMDA oder AMPA receptors or directly via antibody binding to GABA_A_ or GABA_B_ receptors – as a major consequence of humoral neuron-directed autoimmunity in different forms of limbic encephalitis. Moreover, cellular immune effects may either directly or indirectly selectively target inhibitory interneuron networks with similar functional consequences (Figure 1; Table 1).

In other forms of limbic encephalitis, humoral immune effects may predominantly affect excitatory synaptic transmission (and plasticity). Furthermore, cellular immune effects may directly target excitatory principal neuron networks with similar consequences (Figure 1; Table 1).

In the following, we speculate on the consequences of humoral and cellular autoimmune impact on either inhibitory or excitatory pathways for amygdala function, i.e., emotion processing and behavioral responses.

## Putative Effects of Cellular and Humoral Autoimmunity toward Circuit Function of the Amygdala

The local network in the BLA is organized to integrate sensory and affective information and to control emotional responses and cognitive function through its efferent connections to the brainstem, hypothalamus, and forebrain. Key features of the local network function are the strong GABAergic control of activity and the propensity to develop network oscillations at different frequencies. On the one hand, this allows the BLA to resonate with cortical brain regions during emotional information processing; on the other hand, these characteristics may explain why the BLA is prone to the development of epileptic activity.

T cell responses toward GAD65-expressing GABAergic interneurons and autoantibodies to GABA receptors, by reducing GABAergic tone, can be expected to exert profound effects on the amygdala, leading to hyperexcitability of principal cells and a state of increased anxiety and hyperarousal (Figure 1; Table 1).

Loss of GAD65 in knockout mice and concomitant hyperactivity in the amygdala results in anxiety, hyperactivity, and generalized fear (80, 81, 105, 106). It also increases the susceptibility to seizures (107). In fact, it has also been shown that GAD65 antibodies isolated from a stiff-person-syndrome patient induce anxiety in rats upon binding to the amygdala, hippocampus, and prefrontal cortex (108). However, a considerable ability exists to compensate for reduction in GAD65 expression without emergence of profound fear phenotype (109), likely due to activity-dependent regulation of residual enzyme and the co-expression of its isozyme, GAD67, or other components of the GABA metabolism and synaptic cycling. Thus, a considerable variability can be expected in patients with autoimmunity toward GAD65 in interneurons concerning the development of BLA-related symptoms.

Several other autoantibodies also may have the capacity to disturb the inhibitory control of the BLA. For example, loss of GABA_B_ receptors upon antibody binding would likely disrupt synaptic regulation of GABA release in the BLA, which has been related to fear generalization (110). On the post-synaptic level, endocytosis of β3 subunits of the GABA_A_ receptor has been observed during the reinstatement of extinguished fear memory (111). Thus, autoantibody-induced endocytosis of this receptor subunit in the amygdala could have the potential to reactivate memory for fearful experiences, triggering stress and anxiety.

As discussed above, T cell responses toward glutamatergic Hu-expressing principal neurons and antibody-mediated loss of NMDA and AMPA glutamate receptors may affect both glutamatergic and – by mechanisms of homeostatic plasticity – GABAergic neurons in the BLA. Given the local network architecture, it can be expected that this will lead to a reduced net activation of principal cells in the BLA, potentially resulting in disturbed processing of emotional stimuli. Indeed, loss of Hu in knockout mice is associated with lower levels of anxiety and overall activity compared to wild-type counterparts (67). Moreover, lack of precision in information processing in cortico-amygdala circuits can lead to generalization of fear (112) as mechanisms of synaptic competition in the LA contribute to the specificity of conditioned fear (113). GABAergic neurons in the amygdala can be divided into two major populations: cells, which are scatter distributed within the local neurophil (32), and groups of highly clustered GABAergic neurons, the so-called intercalated cells (ITCs) that are targeted by glutamatergic projections from the medial prefrontal cortex mediating fear extinction (114) have been shown to control the information flow between BLA and CeA. Increased internalization of AMPA receptors has been reported in ITCs to mediate long-term depression (LTD) (115). Reduced excitability and plasticity in this structure is likely relevant for the management of previously acquired fearful memories. Hence, internalization of AMPA receptors, induced by AMPA receptor or LGI1 autoantibodies, may mimic a state of LTD in the amygdala (116).

Evidence suggests that autoantibodies to LGI1 are also associated with neuropsychiatric disturbances and seizures (117, 118). In line, selective genetic ablation of LGI1 in excitatory neurons induces seizures, whereas the conditional knockout in parvalbumin expressing interneurons remains without such effect (102). Interfering with the function of LGI1 released from BLA principle cells, via interaction with the disintegrin ADAM23 could alter neuronal morphology and decrease seizure threshold (119), and via ADAM22 reduce the expression of synaptic AMPA receptors (101). On the other hand, somatostatin-positive interneurons in the amygdaloid complex revealed selective vulnerability in temporal lobe epilepsy and following toxic insults, thereby contributing to hyperexcitability of amygdala synaptic circuits and anxiety-like behavior (120–122). Some neuropsychiatric conditions, like schizophrenia, are characterized by remodeling of the perineuronal nets surrounding somatostatin expressing interneurons in the amygdala, which results in GABAergic dysfunction and immune system dysregulation (123), thereby also pointing to possible localized pathological processes in the amygdala.

Moreover, reduced modulatory input from the BLA impairs synaptic plasticity in the hippocampus- and hippocampus-dependent learning. Thus, in addition to putative direct effects on hippocampal synaptic function, the BLA may be involved in memory deficits observed in limbic encephalitis patients. The amygdala provides a site for the modulation of memory acquisition, storage, recall, and modification via the hippocampus (124) and interaction of the BLA with the nAcc is required for active avoidance learning (125). Patients with autoantibodies to voltage-gated K^+^ channels display deficits in cognitive and executive function (126). However, these disturbances of memory-related functions are rather likely to involve direct effects of the autoantibodies on the different amygdala target areas, and at this time it is difficult to estimate the contribution of the BLA to the observed deficits.

The findings discussed above open up the possibility to experimentally assess the role of distinct neuronal cell populations using gene targeting and optogenetics. Furthermore, it can be tested whether disruption of the extracellular matrix, which normally can act as a passive diffusion barrier for cell surface molecules (127) and possibly limits access of immune cells and antibodies to the neuronal cell surface, may influence the impact of both immune effector arms on neuronal network structure and function.

Hence, accumulating evidence suggests that adaptive autoimmune amygdala inflammation may be a major determinant of emotional and behavioral disturbance in limbic encephalitis.

## Conflict of Interest Statement

The authors declare that the research was conducted in the absence of any commercial or financial relationships that could be construed as a potential conflict of interest.
